# Tannin coordinated nanozyme composite-based hybrid hydrogel eye drops for prophylactic treatment of multidrug-resistant *Pseudomonas aeruginosa* keratitis

**DOI:** 10.1186/s12951-022-01653-w

**Published:** 2022-10-14

**Authors:** Hongwei Wang, Fangying Song, Jing Feng, Xia Qi, Li Ma, Lixin Xie, Weiyun Shi, Qingjun Zhou

**Affiliations:** State Key Laboratory Cultivation Base, Shandong Provincial Key Laboratory of Ophthalmology, Eye Institute of Shandong First Medical University, Qingdao, 266071 China

**Keywords:** Tannin-coordinated nanozymes, Hydrogel eye drops, *P. aeruginosa* infections, Multidrug-resistant, Keratitis

## Abstract

*Pseudomonas aeruginosa* infection is a severe acute suppurative ulcer that engulfs virtually the entire tissue in a short period and leads to devastating destruction. Antibiotic therapy is a common approach for the prophylaxis and treatment of *P. aeruginosa* infection. However, it is often associated with serious side effects, complications, and multidrug resistance. Therefore, it has been a long-standing challenge to explore safe and effective methods for controlling *P. aeruginosa* infection. Herein, tannin-coordinated nanozyme composite-based hybrid hydrogels (TCNH) are developed and characterized for the prophylactic treatment of *P. aeruginosa* and multidrug-resistant *P. aeruginosa* infections using mouse keratitis as the animal model. The TCNH eye drops are constructed by photoinitiated free radical polymerization of acetylated gelatin solution containing self-synthesized tannin-coordinated Co_3_O_4_/Ag nanozyme composite. The as-prepared TCNH displays good dispersibility, peroxidase-like activity and in vitro/in vivo biocompatibility. The nanozyme composite in TCNH seems to penetrate the interior of bacteria and exhibited significant broad-spectrum antibacterial activity owing to its intrinsic and nanozymic catalytic properties. Furthermore, TCNH eye drops can be successfully applied to treat *P. aeruginosa* and multidrug-resistant *P. aeruginosa* keratitis. The findings of this study reveal the potential of tannin-coordinated nanozyme composite-based hybrid hydrogel eye drops for treating infectious diseases.

**Graphical Abstract:**

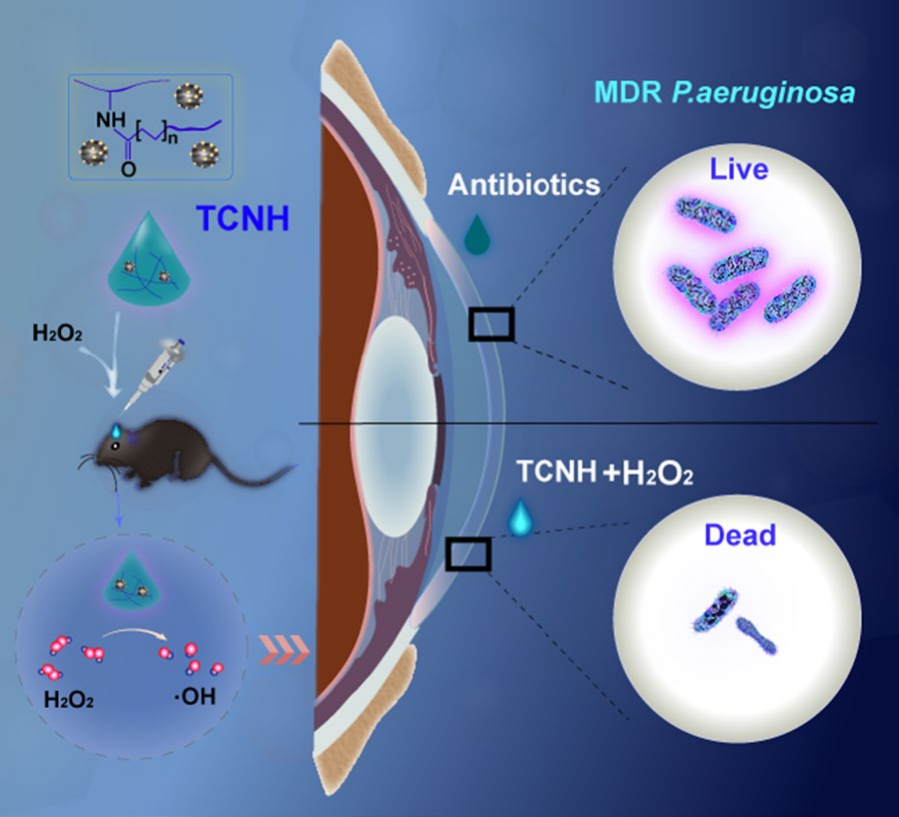

**Supplementary Information:**

The online version contains supplementary material available at 10.1186/s12951-022-01653-w.

## Introduction

Bacterial infections, one of the main problems that threaten public health, are spreading all over the world [1–3]. As common antibacterial agents, there has been considerable success in the treatment of bacterial infections using antibiotics by targeting the processes necessary for bacterial growth and interfering with the synthesis of the cell wall, DNA replication or ribosomal translation [4]. However, bacteria can resist damage by forming biofilms, reducing cell membrane permeability, and expressing efflux pump proteins, which results in the emergence of drug-resistant or even multidrug-resistant (MDR) bacteria and significant reduction in the therapeutic effect of antibiotics [4–6]. Currently, it is estimated that at least 700,000 people worldwide die from bacterial resistance annually [7]. Thus, it remains urgent and challenging to develop novel antibacterial strategies to control drug-resistant infections [3, 7, 8].

Nanozymes including metals, metal oxides, carbon-based materials, and single-atom nanocatalysts are emerging as biomimic nanomaterials [9–12]. With the characteristics of low cost, easy synthesis, recyclability, and high stability, nanozymes can be used to construct a series of antibacterial systems based on their peroxidase-like properties, which convert H_2_O_2_ into highly oxidized hydroxyl radicals (·OH), and effectively inhibit the growth of drug-resistant bacteria [13–15]. At present, metal and metal oxide nanozymes are commonly used for antibacterial applications [7, 16]. However, most of them exhibit low ROS generation capacity and low catalytic activity, which limits their antibacterial efficiencies [17–19]. Currently, nanozyme composites are emerging as an alternative for achieving high ROS generation and improving antibacterial performance compared with metal and metal oxide nanozymes, which can greatly reduce the risk of bacterial resistance [20].

Nanozymes have been employed to treat bacterial infection induced by various common and drug-resistant bacteria such as MRSA *Staphylococcus aureus*, MDR *Escherichia coli*, *Helicobacter pylori*, *Streptococcus pneumoniae* and *Serratia* induced infections [20–25]. Currently, nanozymes have been used for evaluating the antibacterial activity against *Pseudomonas aeruginosa *in vitro [1, 23, 26]. However, they have not been used for the treatment of *P. aeruginosa* or drug-resistant *P. aeruginosa* infectious in vivo, which affect a wide range of patients with traumatized corneas, burns, catheters, cystic fibrosis, and other lung diseases [27, 28]. In addition, nanozymes have been applied to treat bacterial infections and promote wound healing in the skin and stomach, although they have not been used in the treatment of ophthalmic infections [24, 29, 30].

Gelatin hydrogels, possessing water-rich gel networks, are prepared by crosslinking of natural biological collagen hydrolysates of the extracellular matrix and have been receiving intensive attention in the fields of regenerative medicine, tissue engineering, and drug delivery due to their merits such as high hydrophilicity, high swelling degree, excellent biocompatibility, good optical property, and low immune response [31]. Photoinitiated crosslinking polymerization is a simple and efficient approach for the construction of gelatin hydrogels with distinct viscosities by adjusting the precursor concentration and crosslinking time. This renders the homogeneous dispersion of nanoparticles in gelatin hydrogels feasible, avoiding the drawbacks of nanoparticle suspensions including excessive sedimentation and aggregation, and enhancing the catalytic efficiency [32, 33]. In addition, the incorporation of nanoparticles into gelatin hydrogels can improve nanoparticle biocompatibility [34–37]. Thus, UV crosslinking could be a potential approach for the incorporation of nanozymes into gelatin hydrogels and the preparation of safe and effective nanozyme hybrid hydrogels to protect against *P. aeruginosa* infections.

**Scheme 1.**
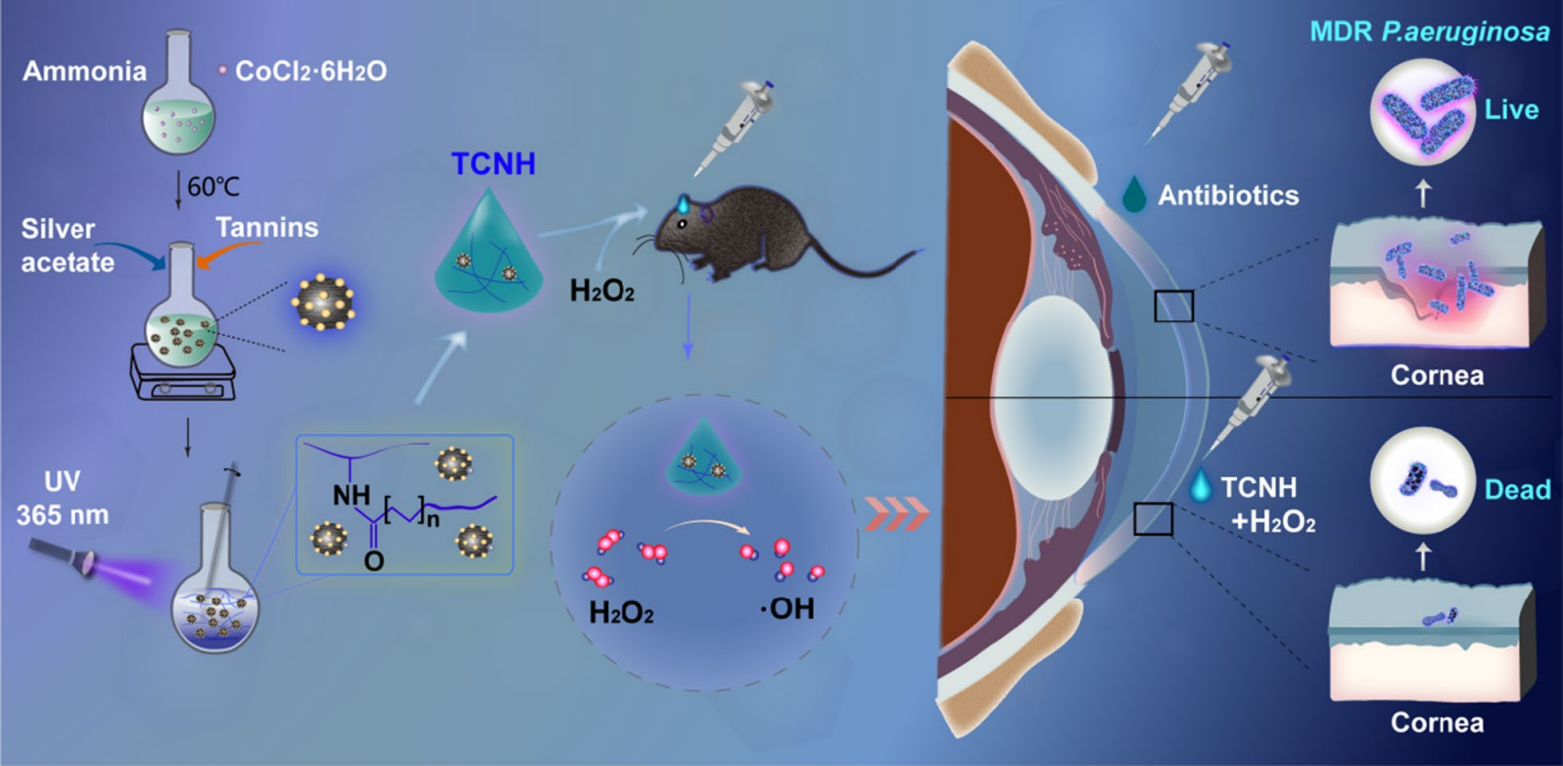
Schematic illustration of tannin-coordinated nanozyme composite-based hybrid hydrogel eye drops for prophylaxis of MDR *P. aeruginosa* keratitis

*P. aeruginosa* keratitis is a special bacterial infection in the cornea that rapidly destroys the entire cornea and induces severe complications including corneal perforation, anophthalmia, and blindness [38–40]. Without prompt intervention, the eyeballs would be irreparably extirpated. Therefore, it is of great urgency to develop safe, effective, and antibiotic-independent approaches to treat *P*. *aeruginosa* keratitis. In this study, we aimed to construct tannin-coordinated nanozyme-based hybrid hydrogel (TCNH) eye drops for prophylactic treatment of *P. aeruginosa* keratitis (Scheme 1). The TCNH eye drops were prepared in two steps. First, tannin-coordinated nanozyme (TCN) was fabricated by hydrothermal synthesis of cobalt tetroxide nanoparticles, metal-polyphenol coordination for immobilizing tannin into cobalt tetroxide and chemical reduction of silver nitrate for post-decoration of silver nanoparticles on the surface of cobalt tetroxide. Then, the resulting TCN nanoparticles were suspended in gelatin solution, and the mixture was further crosslinked by photoinitiated free radical polymerization. The physical characteristics, peroxidase-like activity, in vitro/in vivo biocompatibility, and in vitro antibacterial activity of the resulting TCNH eye drops were evaluated. Thereafter, we further assessed the efficacy of the eye drops in the prophylactic treatment of *P. aeruginosa* and MDR *P. aeruginosa* keratitis.

## Materials and methods

### Chemicals

CoCl_2_·6H_2_O, tannin, silver acetate, 3,3',5,5'-tetramethylbenzidine (TMB), *o*-phenylenediamine (OPD) and gelatin were obtained from Macklin Biochemical Co., Ltd. (Shanghai, China). Acrylic anhydride was purchased from Sigma (St Louis, MO, USA). Hydrogen peroxide (H_2_O_2,_ 30%), aqueous ammonia, acetic acid and ethanol were the products of Tianjin Kermel Chemical Plant (Tianjin, China). 2,2-Dimethoxy-2-phenylacetophenone (DMPA), the live/dead (SYTO 9/PI) bacterial viability kit and PE anti-mouse CD45 (Cat# 12-0451-82) were received from Thermo Fisher Scientific (New Jersey, USA). The lactate dehydrogenase (LDH) cytotoxicity assay kit was obtained from Beyotime (Shanghai, China). 2',7'-Dichlorodihydrofluorescein diacetate (DCFH-DA) was obtained from Solarbio Life Science (Beijing, China). FITC anti-mouse F4/80 (Cat# 123108) and FITC anti-mouse Ly-6C/Ly-6C (Gr-1) (Cat# 108406) were obtained from Biolegend (San Diego, CA, USA). Anti-Ly6G + Ly6c (ab25377) and anti F4/80 (ab111101) were purchased from Abcam (Cambridge, UK). GTVisionTM I detection system/Mo&Rb (GK500510A) was received from Gene Tech (Shanghai) company limited. Four strains of MDR *P. aeruginosa* (21027, 20059, 21173, 21715) were kindly provided by Qingdao Municipal Hospital (China). Doubly deionized water was purified by a Unique-R20 purification system (Xiamen, China).

### Preparation of TCNH

TCN was prepared using the following procedure. Aqueous ammonia (25%, 1.0 mL) was slowly dripped into a CoCl_2_·6H_2_O solution (20.0 mmol L^−1^, 50.0 mL). The mixture was vigorously stirred for 10 min, and heated to 60 °C, followed by the sequential addition of H_2_O_2_ (30%, 60.0 μL) solution and CoCl_2_·6H_2_O solution (25.0 mmol L^−1^, 20.0 mL). After cooling to room temperature, tannin solution (58.5 mg/mL, 40.0 mL) and silver nitrate aqueous ammonia (25.0 mg/mL, 10.0 mL) were added, and the mixture was further stirred for 16 h. The product was centrifuged at 10,000 rpm for 10 min, extensively washed with ethanol, and dried at 60 °C in a vacuum oven for 4 h. The sample was calcined at 400 °C under nitrogen for 2 h to obtain TCN.

TCN (40.0 mg), 10% DMPA ethanol solution (10.0 µL), and 5% acrylated gelatin solution (10.0 mL) were mixed in a 50 mL round bottom flask. The mixture was stirred at room temperature for 15 min, and irradiated under ultraviolet light (365 nm) for 30 min to obtain TCNH.

### Material characterizations

Scanning electron microscopy (SEM) and transmission electron microscopy (TEM) images of the as-prepared samples were recorded on a Gemini SEM 300 scanning electron microscope (Zeiss, Oberkochen, Germany) and a JEOL 2000 EX electronic microscope (JEOL, Tokyo, Japan), respectively. High angle annular dark-field scanning TEM (HAADF-STEM) and energy dispersive X-ray spectroscopy (EDS) mapping images were collected on a FEI Talos F200X TEM electron microscope at 200 kV (Thermo Fisher Scientific, USA). The X-ray photoelectron spectroscopy (XPS) spectra were collected on an ESCALAB 250 photoelectron spectrometer (Thermo-VG Scientific, USA) using Al Ka (1486.6 eV) as the X-ray source. Powder X-ray powder diffraction (PXRD) of the sample was performed on an X-ray powder diffractometer using CuKa radiation. The surface areas of the samples were calculated from nitrogen adsorption/desorption measurements on a Quadrasorb SI4 adsorptometer (Quantachrome, Boynton Beach, USA). Fourier-transform infrared spectroscopy (FT-IR) characterization was performed on a Thermo Nicolet 380 spectrometer using KBr pellets (Nicolet, Wisconsin, USA). Hydroxyl radicals were measured using a JEOL JES-FA200 electron spin resonance (ESR) spectrometer.

### The peroxidase-like property of TCNH

The peroxidase-like activity of TCNH was evaluated based on the catalytic oxidation reaction of the substrate TMB into oxTMB in the presence of H_2_O_2_. In brief, H_2_O_2_ (20.0 μL, 0.5 M) were added to PBS solution (pH 4.0, 1.0 mL) containing TMB (10.0 μL, 16.0 mM) and TCNH solution (4.0 mg/mL, 5.0 μL) to start the catalytic process. The reaction was monitored by measuring the UV–vis absorbance of oxTMB at 652 nm using a NanoDrop™ One UV–vis spectrophotometer (Thermo Fisher Scientific, USA). In addition, pH-dependent (2.0–12.0) and temperature-dependent (4, 25, 37, and 60 °C) experiments were utilized to study the influence of pH and temperature on the catalytic activity of TCNH. All tests were performed in triplicate. OPD was also adopted to characterize the peroxidase-like activity of TCNH with a similar method as TMB for the UV–vis absorbance of the product 2,3-diaminophenazine (DAP) at 450 nm.

### Biocompatibility evaluation

Immortalized human corneal epithelial cells (HCECs) and human corneal fibroblasts (HCFs) were adopted to evaluate the cytotoxicity of TCNH using a lactate dehydrogenase (LDH) cytotoxicity assay kit [37]. In brief, cells with densities of 1 × 10^4^ were seeded on 96-well plates, cultured in DMEM/F12 medium supplemented with 10% (v/v) FBS and 1% (v/v) penicillin/streptomycin, and incubated at 37 °C in a 5% CO_2_ atmosphere for 24 h. Afterwards, TCNH (1.0 μL) was added to the culture (1.0 mL), and 200.0 μL of the mixture was removed and added to the 96-well plates. The cells were incubated for another 24 h. After centrifugation at 1000 rpm for 5 min, the supernatant was discarded, and 150.0 μL of LDH releasing reagent was added for further incubation for 1 h. Then 120.0 μL of supernatant and 60.0 μL of LDH detection solution were added to another well after centrifugation at 1000 rpm for 5 min and incubated at room temperature in the dark for 30 min. The absorbance was measured at 490 nm. In addition, the cytotoxicity of TCNH in the presence of H_2_O_2_ was evaluated with the same method as that of TCNH.

The effect of TCNH on viability of HCECs and HCFs was examined by a live/ dead cell double staining kit. Briefly, cells with a density of 1 * 10^5^ were grown on glass-bottom cell culture dishes for 24 h, and then cultured with diluted TCNH for another 24 h. The staining solution was prepared by mixing LiveDye (1.0 μL) and NucleiDye (1.0 μL) in DMEM/F12 medium (1.0 mL) supplemented with 10% (v/v) FBS and 1% (v/v) penicillin/streptomycin. Afterwards, the cells were stained at 37 °C for 30 min and washed twice with PBS (pH 7.0) before visualization by CLSM. Live and dead cells were dyed green and red, respectively. In addition, the effect of TCNH in the presence of H_2_O_2_ on the viability of cells was surveyed with the abovementioned method.

The in vivo biocompatibility of TCNH in the presence of H_2_O_2_ was assessed using 8-week-old female C57BL/6J mice. TCNH (4.8 μL) and H_2_O_2_ (10 mM, 0.2 μL) were mixed and dripped onto the mouse corneas once every hour for 8 h. The mice were sacrificed at 48 h post saline and TCNH + H_2_O_2_ intervention. The corneas were carefully harvested for further histopathological examination by hematoxylin–eosin (H&E) staining and morphological observation by SEM.

### Antibacterial assays

The antibacterial activity of TCNH was assessed by the plate cultivation method. In brief, bacterial monocolony and LB culture medium (20.0 mL) were mixed and incubated at 37 °C for 16 h. After centrifugation, *P. aeruginosa* (19,660) was collected and diluted to 2*10^9^ CFU/mL. Then bacterial suspension (1.0 μL), TCNH (191.0 μL), and H_2_O_2_ (10 mM, 8.0 μL) were mixed and further incubated at 180 rpm at 37 °C for 30 min. The mixture was diluted by gradient dilution, spread onto the LB agar plates, and then cultured at 37 °C overnight to count the bacterial loads. In addition, the mixture was also stained with a live/dead (SYTO 9/PI) bacterial viability kit for fluorescence detection and fixed with 2.5% glutaraldehyde for SEM analysis. Using the abovementioned method, *E. coli* (25922) and *Candida albicans* (98001) were adopted to evaluate the broad-spectrum antibacterial performance of TCNH, and the antibacterial activity of TCNH against drug-resistant bacteria was investigated using four strains of MDR *P. aeruginosa* (21027, 20059, 21173, 21715).

The anti-biofilm activity of TCNH was investigated using the crystal violet (CV) staining method. In brief, TCNH (8.0 mg/mL, 500.0 μL), H_2_O_2_ (10 mM, 40.0 μL), LB medium (455.0 μL) and bacterial suspension (2*10^9^ CFU/mL, 5.0 μL) were added to 48-well plates, and the mixture was cultured at 37 °C for 48 h. The formed biofilms were washed with PBS (pH 7.0) and stained with 1% CV staining solution for 20 min. The stained biofilms were then washed three times gently to remove unbonded CV and naturally dried. Acetic acid was used to dissolve the attached CV for quantitative analysis of the formed biofilms. The absorbance was measured using a microplate reader at 595 nm.

The biofilm was further cultured on a round glass slide with a similar procedure as above. After 48 h, the glass slides were gently removed and washed with PBS (pH 7.0) to remove planktonic bacteria. Then the slides were stained with a live/dead (SYTO 9/PI) bacterial viability kit and observed under a confocal laser scanning microscope (CLSM).

### Intracellular ROS detection

Intracellular ROS levels were determined by fluorescence spectroscopy using the 2′,7′-dichlorodihydrofluorescein diacetate (DCFH-DA) method. Briefly, the bacterial suspension (2*10^9^ CFU/mL, 1.0 mL) was incubated with DCFH-DA (10.0 μL) for 30 min and centrifuged at 5000 rpm for 10 min. The sediments were washed with PBS (pH 7.0) 3 times to remove the free DCFH-DA. The collected bacteria were further used for conducting antibacterial experiments using the same procedure as in *Biocompatibility Evaluation* part. The resulting mixtures were then photographed with a fluorescence microscope (Nikon, Japan).

### *Pseudomonas aeruginosa* keratitis models in mice

All animal experiments were approved by the Shandong Eye Institute ethics committee and were carried out based on the ARVO Statement for the Use of Animals in Ophthalmic and Vision Research. C57BL/6J mice (8 weeks old, female) were purchased from Beijing Vital River Laboratory Animal Technology Co., Ltd. (Beijing, China). The *P. aeruginosa* (19660) keratitis model was selected to evaluate the antibacterial efficacy of TCNH in vivo. Mice (*n* = 32) were anesthetized through intraperitoneal injection of 0.6% pentobarbital (30.0 mg/kg). Afterwards, three 1-mm incisions were created on mouse corneas using a sterile 25-gauge needle and the mice were randomly divided into four groups (*n* = 8). The wound surface was inoculated with *P. aeruginosa* (5.0 μL, 10^5^ CFU), and the mice in the four groups were treated with saline (5.0 μL), H_2_O_2_ (5.0 μL), TCNH (5.0 μL), or TCNH + H_2_O_2_ (5.0 μL), once every hour for a total of 8 doses. The corneas were observed using a slit lamp at 24 and 48 h to detect the infection progression. After 48 h of the first administration, all mice were sacrificed and the eyeballs were harvested for H&E staining, SEM, flow cytometry, bacterial load counting, and qRT–PCR analysis.

In addition, *P. aeruginosa* (21027) was selected to evaluate the antibacterial efficacy of TCNH on MDR *P. aeruginosa* keratitis in mice. Mice (*n* = 32) were randomly divided into four groups (*n* = 8): saline, tobramycin (0.3 mg/mL), gentamicin (0.3 mg/mL), and TCNH + H_2_O_2_. Other experimental processes were performed with the same procedure as that for *P. aeruginosa* (19,660) keratitis.

### Flow cytometry

The mouse eyeballs in the four groups were collected, placed in 96-well plates, and digested overnight at 4 °C with dispase (15.0 mg/mL). The corneal epithelium and stromal layer, separated under a microscope and placed separately, were digested with trypsin (0.25%) and collagenase (2.0 mg/mL) at 37 °C for 1–2 h, respectively. The suspensions were merged, incubated with antibody mixtures (Ly6G-PE, F4/80-PE) for 30 min on ice, and further surveyed using a flow cytometer (BD FACSCalibur, USA).

### Histopathological analysis

The harvested eyeballs were fixed in 10% neutral buffered formalin for 24 h, embedded in paraffin and cut into slices (5 μm-thickness). The slices were stained with H&E, and further imaged under a light microscope (Nikon, Japan). For immunohistochemistry, tissue slices were stained with primary antibodies (anti-Ly6G + Ly6c, anti F4/80), and subsequently reacted with secondary antibody (GTVisionTM I detection system/Mo&Rb). The hematoxylin was adopted to visualize the nuclei. The staining was analyzed under a light microscope (Nikon, Japan).

### Statistical analysis

All tests were performed at least three times. The data were statistically analyzed using Student’s t tests by Prism 8 (GraphPad, San Diego, CA, USA). A value of *P < 0.05 was considered statistically significant between the means. The results are presented as the mean ± standard deviation (SD).

## Results and discussion

### Fabrication and characterization

**Fig. 1:**
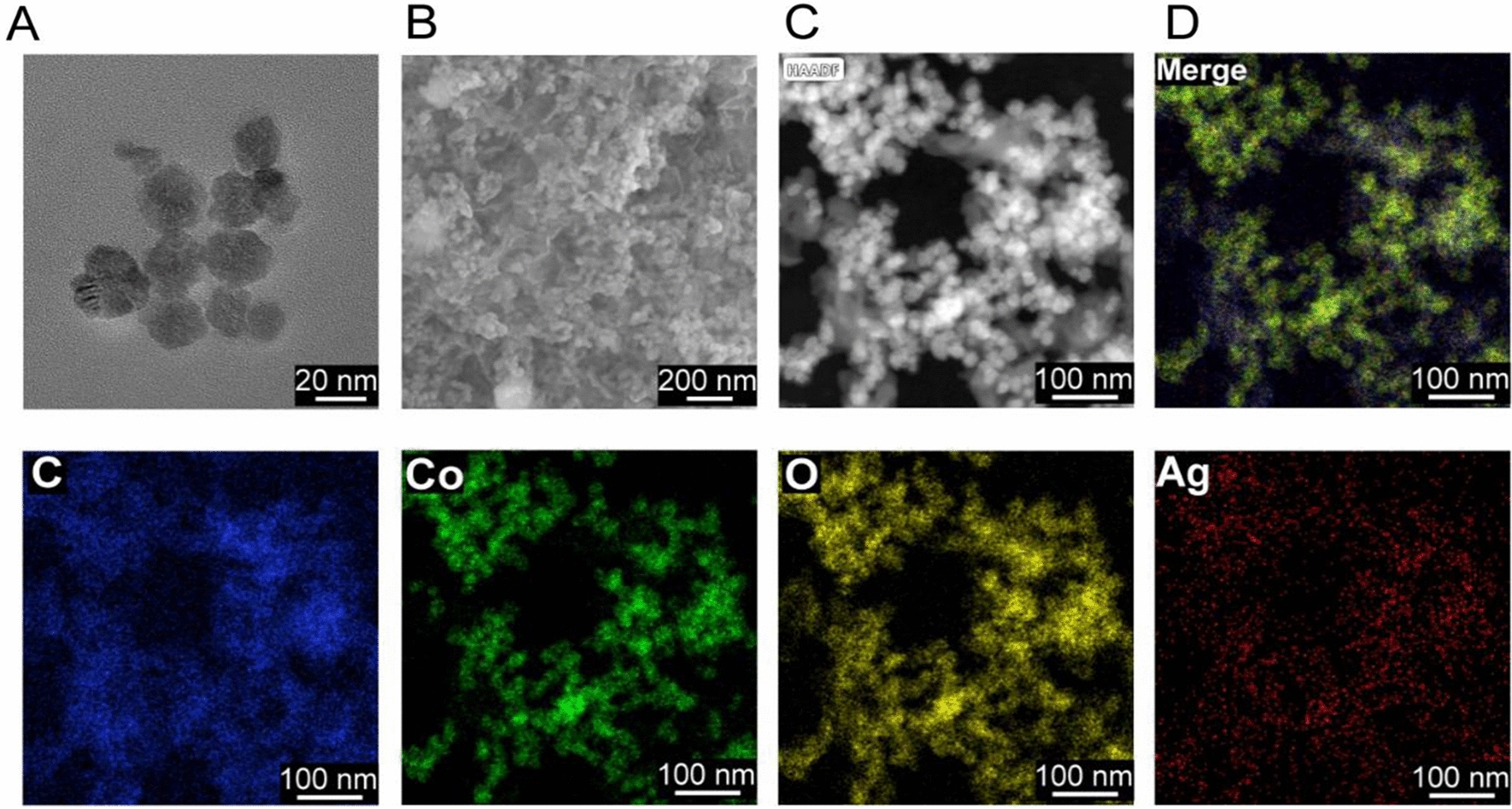
Morphology characterizations. TEM **A**, SEM **B** of TCN; HAADF-STEM image of TCN **C**, HAADF-STEM-EDS elemental mapping images of Co, Ag, C, and O in TCN **D**

**Fig. 2:**
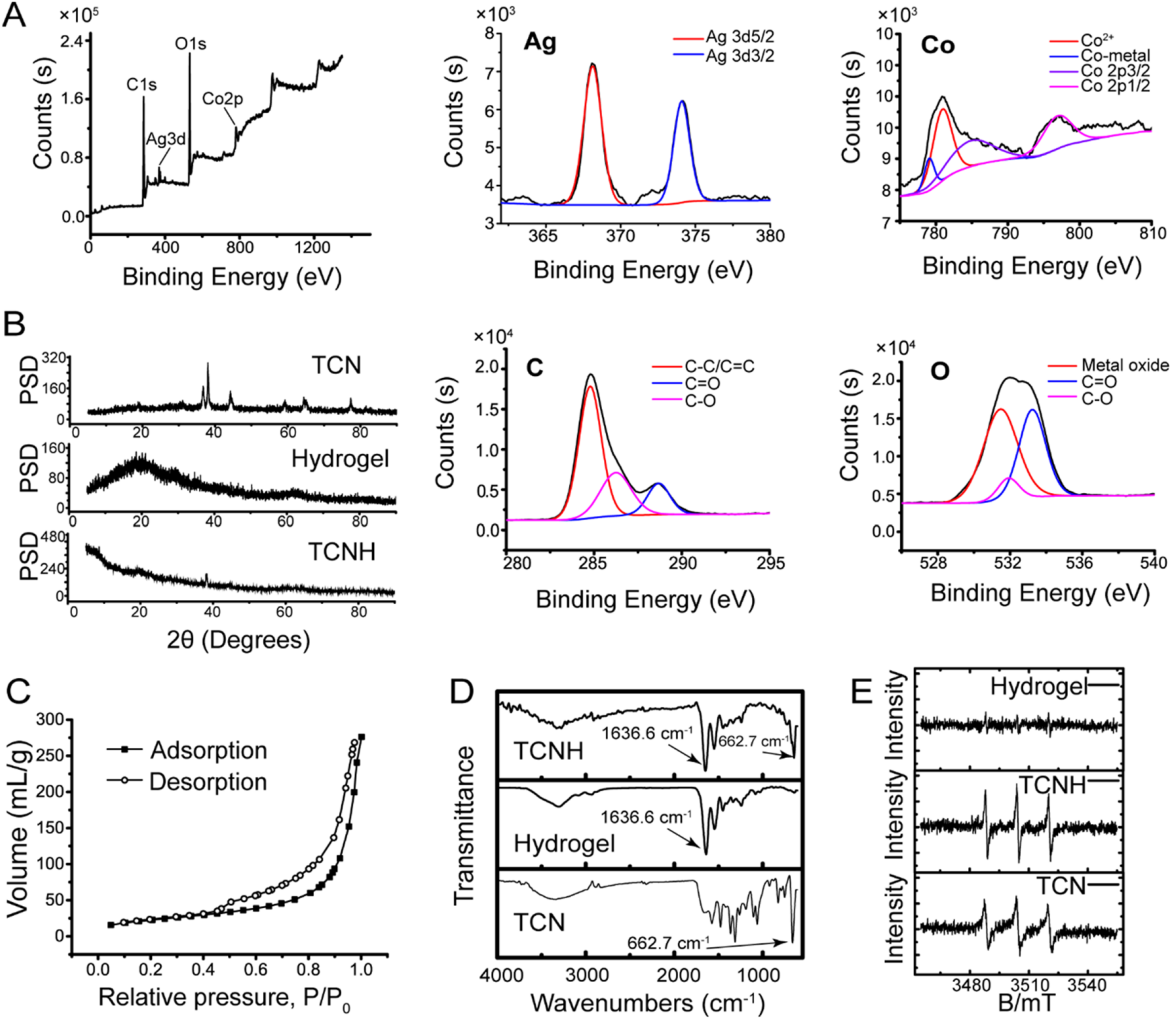
Structural and property characterizations. XPS spectra of TCN **A**; PXRD spectra of hydrogel, TCN, and TCNH **B**; N_2_ adsorption/desorption isotherms of TCN **C**; FT-IR spectra of hydrogel, TCN, and TCNH **D**; ESR spectra of hydrogel, TCN, and TCNH in the presence of H_2_O_2_ **E**

In the study, TCN was constructed by hydrothermal synthesis of cobalt tetroxide nanoparticles, metal-polyphenol coordination for immobilizing tannin into cobalt tetroxide and chemical reduction of silver nitrate for post-decoration of silver nanoparticles on the surface of cobalt tetroxide. Then, the TCNH eye drops were obtained by photoinitiated free radical polymerization of acetylated gelatin suspended with the as-prepared TCN nanoparticles. The morphology, structural properties, and chemical compositions of TCN were characterized by TEM and SEM. The images indicated the nanoscale spherical structure of TCN and the macroporous structure of TCNH with dozens of micrometer-sized pores (Fig. 1A, B, Additional file 1: Figure S1). HAADF-STEM, EDS mapping images and EDS analysis (Fig. 1C, D, Additional file 1: Figure S2) presented Ag, C, Co, and O elements in TCN. XPS was adopted to investigate the chemical compositions and electronic states of TCN. The XPS survey spectrum (Fig. 2A) provided typical peaks of Ag, Co, C1s, and O1s. In the high-resolution XPS spectrum of Ag, the peaks at 374.1 and 368.2 eV were ascribed to Ag 3d3/2 and Ag 3d5/2, respectively, which was in accordance with the electron-binding energies of Ag 3d. In the Co spectrum of TCN, the peaks at 797.2, 785.2, 781.0, and 779.1 eV were assigned to Co 2p1/2, Co 2p3/2, Co^2+^, and Co-metal, respectively. In addition, the high-resolution C1s XPS spectrum exhibited the presence of C = C/C–C (284.5 eV), C = O (288.5 eV), and C-O (286.5 eV) groups. The O1s XPS spectrum could be deconvoluted into three peaks at 533.5 eV (C = O), 532.5 (C-O), and 531.5 eV (metal-oxide). These results confirmed the coordination of tannin with the Co_3_O_4_/Ag composite in TCNs. PXRD was then used to determine the crystallographic structure of the hydrogel, TCN and TCNH (Fig. 2B). The PXRD patterns of TCN exhibited diffraction peaks at 36.8, 44.2, 59.4, 64.8, 77.4, and diffraction peak at 38.0, which corresponded to the spinel cobalt tetroxide and cubic metallic Ag, respectively, while the PXRD pattern of the hydrogel exhibited no signals. After incorporation with TCN, TCNH exhibited a PXRD peak at 38.0, which mainly corresponded to TCN in TCNH.

Nitrogen adsorption-desorption experiments were performed to characterize the specific surface area of TCN using the Brunauer-Emmett-Teller model within the relative pressure range of 0.5–1.0, as shown in Fig. 2C. Type IV isotherm characteristics suggested the typical mesoporous structure of TCN, which could be attributed to nanoparticle packing. The specific surface area of TCN was calculated to be 126.7 m^2^/g. Figure 2D shows the FT-IR spectra of TCN, gelatin hydrogel, and TCNH. The peaks at 3339.4, 1575.1, 1477.0, 1364.9, and 1105.9 cm^−1^ implied the involvement of phenolic hydroxyls, aromatic rings (C = C stretching), and C-O bonds in TCN, manifesting the immobilization of tannin into cobalt tetroxide [41]. In addition, the characteristic signals of amino groups (1636.6 cm^−1^) in gelatin hydrogel and Co^III^–O vibration (662.7 cm^−1^) in TCN were present in the spectrum of TCNH, indicating the successful incorporation of TCN into the hydrogel. In addition, ·OH signals were monitored by ESR. As shown in Fig. 2E,·OH signals were detected in the TCN + H_2_O_2_ and TCNH + H_2_O_2_ groups, but not in the hydrogel + H_2_O_2_ group, confirming the generation of ·OH by H_2_O_2_ under the catalysis of TCN. These results indicated the successful synthesis of TCN and TCNH.

Noteworthily, several tannic acid modified nanoparticle systems such as Fe^2+^-tannic acid, Fe^3+^-tannic acid and Ag-tannic acid have been developed for in vivo disease treatments [42–45]. Compared with these systems, the TCN nanoparticle in the TCNH system can be homogeneously dispersed in gelatin hydrogels, avoiding the disadvantages of nanoparticle suspensions including excessive sedimentation and aggregation, which could provide a support for the satisfactory peroxidase-like activity, biocompatibility, and antibacterial performance.

### Peroxidase-like activity

**Fig. 3:**
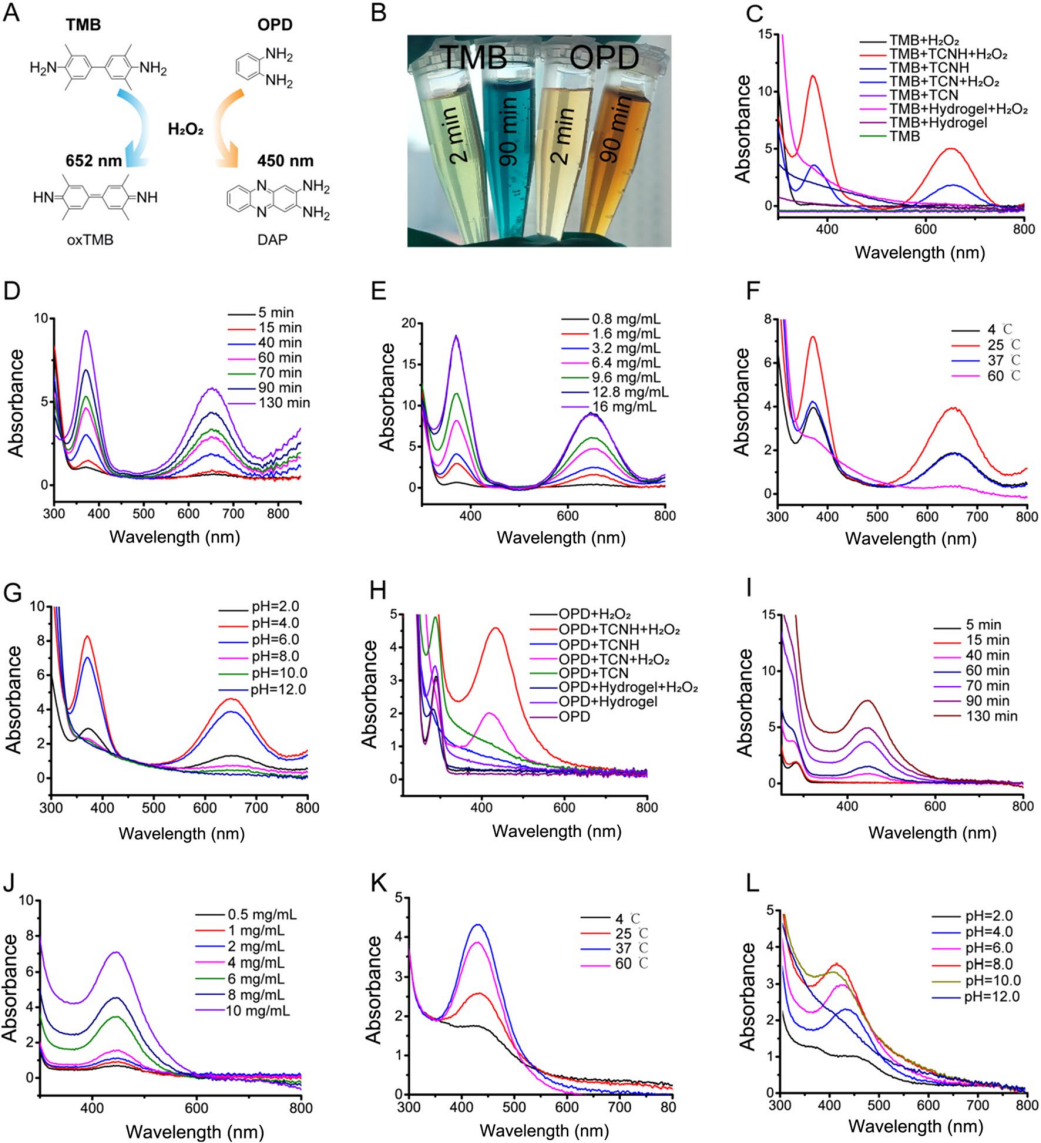
Peroxidase-like performance of TCNH using TMB and OPD probes. Types of TMB and OPD oxidations **A**. Digital photographs of TMB and OPD oxidations **B**. TMB reactions at different compositions of reaction systems **C**, reaction times **D**, TMB concentrations **E**, reaction temperatures **F**, and pH values **G**. OPD reactions at different compositions of reaction systems **H**, reaction times **I**, OPD concentrations **J**, reaction temperatures **K**, and pH values **L**

The peroxidase-like activity of TCNH was spectroscopically evaluated using 3,3',5,5'-tetramethylbenzidine (TMB) and H_2_O_2_ as substrates. ·OH was produced by co-incubation of H_2_O_2_ with TCNH, which oxidized TMB into ox-TMB and changed the solution color from colorless to blue (Fig. 3A ,B). Blue color with absorption at 652 nm was not produced in the TMB, TMB + H_2_O_2_, TMB + H_2_O_2_ + hydrogel, TMB + TCN, TMB + TCNH, and TMB + hydrogel groups, while both the TCN + H_2_O_2_ + TMB and TCNH + H_2_O_2_ + TMB groups exhibited absorption peaks at 652 nm, suggesting that TCNH and TCN possessed peroxidase-like activities (Fig. 3C). The stronger adsorption peak in the TCNH + H_2_O_2_ + TMB group than in the TCN + H_2_O_2_ + TMB group could be attributed to the good dispersibility of TCN in the hydrogel, which might enhance the catalytic efficacy of TCN. Then the effects of reaction time and TMB concentration on catalytic activity were further investigated (Fig. 3D, E). As the reaction time or TMB concentration increased, the adsorption peaks at 652 nm were intensified, demonstrating the time-dependent and concentration-dependent peroxidase-like catalytic performances. In addition, we further surveyed the effect of temperature on the TMB catalytic activity of TCNH. As shown in Fig. 3F, the intensity of the adsorption peak at 25 °C was stronger than those at 4 °C, 37 °C, and 60 °C. The result was similar with the TMB catalytic activity of Co_3_O_4_-reduced graphene oxide nanocomposite in Shi’s previous report [46]. Furthermore, adsorption peaks of TMB reaction at pH 2.0–12.0 indicated higher peroxidase-like performances at pH 4.0 and 6.0 than those at other pH values (Fig. 3G). In addition, we also evaluated the peroxidase-like catalytic performance of TCNH using *o*-phenylenediamine (OPD) and H_2_O_2_ (Fig. 3A, B). Similarly, only the TCN + H_2_O_2_ + OPD and TCNH + H_2_O_2_ + OPD groups exhibited absorption peaks at 450 nm (Fig. 3H). The time-dependent and concentration-dependent peroxidase-like activities of TCNH were also demonstrated by intensified adsorption peaks at 450 nm with increasing time and OPD concentration (Fig. 3I, J). In addition, the intensity of the adsorption peak increased with increasing temperature from 4 °C to 37 °C, while the intensity slightly decreased with a further increase in temperature to 60 °C (Fig. 3K). Moreover, peroxidase-like performances of TCNH at various pH values were also corroborated using the OPD reaction, which was similar to those using the TMB reaction (Fig. 3L). The TCNH behaviors in the TMB and OPD reactions proved the peroxidase-like catalytic activity of TCNH.

### Biocompatibility assessment

**Fig. 4:**
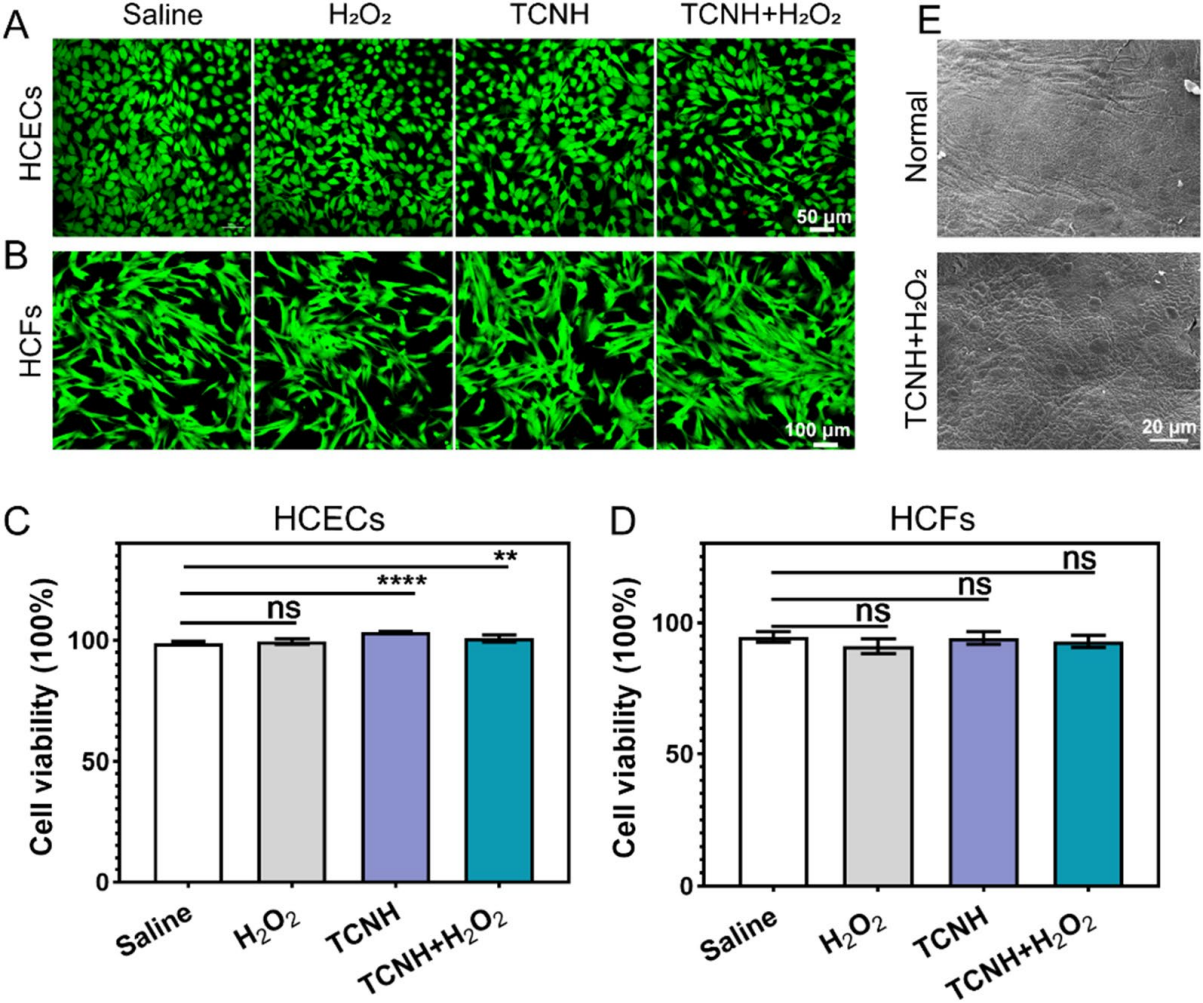
In vitro and in vivo biocompatibility of TCNH. Representative CLSM images of HCECs **A** and HCFs **B** by live/dead cell double staining after 24 h of cultivation. The quantitation of HCEC **C** and HCF **D** viability by LDH cytotoxicity assay kit after saline, H_2_O_2_, TCNH, and TCNH + H_2_O_2_ interventions. Representative SEM images of mouse corneas after saline and TCNH + H_2_O_2_ interventions **E**

The biocompatibility of TCNH was assessed by in vitro and in vivo experiments before investigation of its antibacterial activity. Initially, the effects of TCNH on the viability of HCECs (Fig. 4A, C) and HCFs (Fig. 4B, D) were evaluated via live/dead cell staining experiments. The results showed that the cells were basically dyed green (live) in the four (saline, H_2_O_2_, TCNH, TCNH + H_2_O_2_) groups after 24 h of intervention. Further LDH assays showed that the cell viabilities of HCECs and HCFs in all treated groups remained greater than 90%, demonstrating the nontoxicity of TCNH to cell growth. The in vivo biocompatibility of TCNH in the presence of H_2_O_2_ was verified by dripping TCNH + H_2_O_2_ onto the corneal surface of mice. The corneas at 48 h post intervention were observed by SEM (Fig. 4E). The images of corneal epithelial cells in the TCNH + H_2_O_2_ group showed a similar morphology to that in the saline group, indicating no obvious damage to the corneas after TCNH + H_2_O_2_ intervention. Both in vitro and in vivo experiments suggested the excellent biocompatibility of TCNH.

### In vitro antibacterial performance

The H_2_O_2_ and TCNH concentrations were optimized prior to antibacterial activity evaluation of TCNH against *P. aeruginosa* (19,660). As shown in Additional file 1: Figure S3A, the log_10_CFU numbers of *P. aeruginosa* in both the TCNH + H_2_O_2_ and H_2_O_2_ groups were over 7.0, and there was no obvious difference between the log_10_CFU numbers in two groups when the H_2_O_2_ and TCNH concentrations were set as 100 μM and 0.4 mg/mL, respectively, indicating the poor antibacterial activities of H_2_O_2_ and TCNH + H_2_O_2_ against *P. aeruginosa* under these conditions. With the increase in H_2_O_2_ concentration from 100 μM to 400 μM, log_10_CFU number in the TCNH + H_2_O_2_ group significantly decreased, while those in the H_2_O_2_ group were slightly reduced, indicating the improved antibacterial activity of TCNH + H_2_O_2_ at 400 μM. Then we further investigated the effect of TCN concentration in TCNH on log_10_CFU numbers with H_2_O_2_ concentration at 400 μM (Additional file 1: Figure S3B). With the increase in TCN concentration from 0.4 mg/mL to 2.0 mg/mL, the antibacterial activity of TCNH against *P. aeruginosa* gradually increased. As the TCN concentration increased to 4.0 mg/mL, log_10_CFU number dramatically decreased from 3.0 to 0. Based on these results, H_2_O_2_ (400 μM) and TCN concentration (4.0 mg/mL) were selected for subsequent antibacterial experiments.

**Fig. 5:**
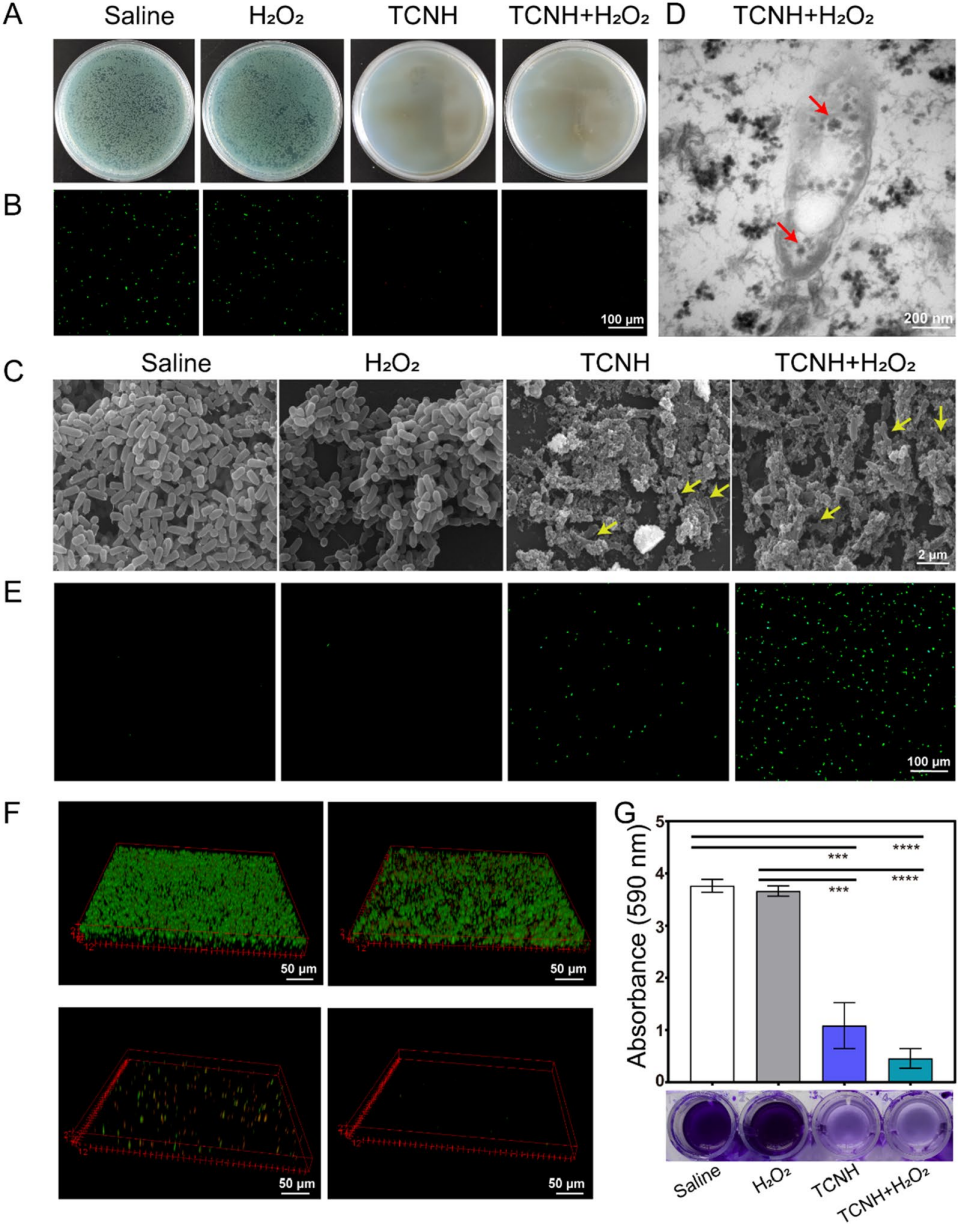
In *vitro* antibacterial activity of TCNH against *P. aeruginosa* (19660). Representative photographs of bacterial colonies **A**, CLSM images of live/dead bacterial staining **B** and SEM images of bacteria **C**. TEM image of bacteria treated with TCNH + H_2_O_2_ **D**. Intracellular ROS levels **E**, CLSM images of biofilms **F** and CV staining assay **G** by saline, H_2_O_2_, TCNH, TCNH + H_2_O_2_ interventions

The antibacterial property of TCNH against *P. aeruginosa* (19,660) was initially evaluated using the plate cultivation method. Figure 5A shows the plate cultivation results of bacteria after different interventions (saline, H_2_O_2_, TCNH, and TCNH + H_2_O_2_). The TCNH + H_2_O_2_ and TCNH groups exhibited high antibacterial activities as nearly no bacterial colonies were observed after interventions. However, bacteria all overgrew the LB agar plate in the saline and H_2_O_2_ groups, and there was no significant difference between two groups. In the dead/live (SYTO 9/PI) double staining assays (Fig. 5B), *P. aeruginosa* exhibited green fluoresce (live) in the saline and H_2_O_2_ groups, indicating that the bacteria in both groups were in good condition. After intervention with TCNH + H_2_O_2_ and TCNH, the numbers of live bacteria in both groups significantly decreased, which was consistent with the plate cultivation results. The incubated mixtures in the four intervention groups were centrifuged and the residues were further observed by SEM. As shown in Fig. 5C, *P. aeruginosa* in the saline treated group presented as a long oval rod with a smooth surface. After treatment with a low concentration of H_2_O_2_, the bacteria maintained their rod shape, and rare disruptions were observed on the bacterial surface, suggesting the poor antibacterial activity of H_2_O_2_ against *P. aeruginosa*. However, the bacterial cells were always accompanied by deformation and dents after intervention with TCNH + H_2_O_2_ and TCNH, indicating the obvious damage to *P. aeruginosa* caused by TCNH + H_2_O_2_ and TCNH. Then ROS levels in the four groups were detected by the DCFH-DA method (Fig. 5E). It was clearly found that there were nearly no ROS in either the saline or H_2_O_2_ group, sporadic ROS in the TCNH group and widely distributed ROS in the TCNH + H_2_O_2_ group. The omnipresent ROS in the TCNH + H_2_O_2_ group could be produced by the reaction of TCNH with exogenous H_2_O_2_. In addition, the morphology of TCNH + H_2_O_2_ treated bacteria was evaluated by TEM (Fig. 5D). The results revealed that TCN could penetrate the interior of *P. aeruginosa* due to its small particle size (red arrow), which might explain the process for inhibiting *P. aeruginosa* using TCNH.

Biofilms, complexes of DNA, proteases, and polysaccharides, are self-produced and encapsulated by bacteria. It can prevent the penetration of antibiotics and reduce the efficacy of antibiotics in the treatment of bacterial infection [21, 37]. Therefore, it was of significance to investigate the antibiofilm capacity of TCNH before evaluating its performance in inhibiting *P. aeruginosa* keratitis. As shown in Fig. 5F, the formed biofilms in the four groups were observed by CLSM after SYTO 9/PI staining. Biofilms of *P. aeruginosa* were well formed after intervention with saline and H_2_O_2_ solution. However, there was no obvious biofilm formation in the TCNH + H_2_O_2_ and TCNH groups, indicating the significant inhibition efficacy of TCNH + H_2_O_2_ and TCNH against *P. aeruginosa* biofilm formation. The biofilms in the four groups were also quantified by CV staining assay (Fig. 5G). Compared with the saline and H_2_O_2_ groups, the TCNH + H_2_O_2_ and TCNH groups exhibited significantly reduced peak intensity (590 nm) and were demonstrated to be effective in inhibiting the formation of *P. aeruginosa* biofilms. The hybrid hydrogel can generate highly toxic ·OH in the presence of H_2_O_2_ due to its peroxidase-like activity, which could account for the antibacterial and antibiofilm performances of TCNH.

**Fig. 6:**
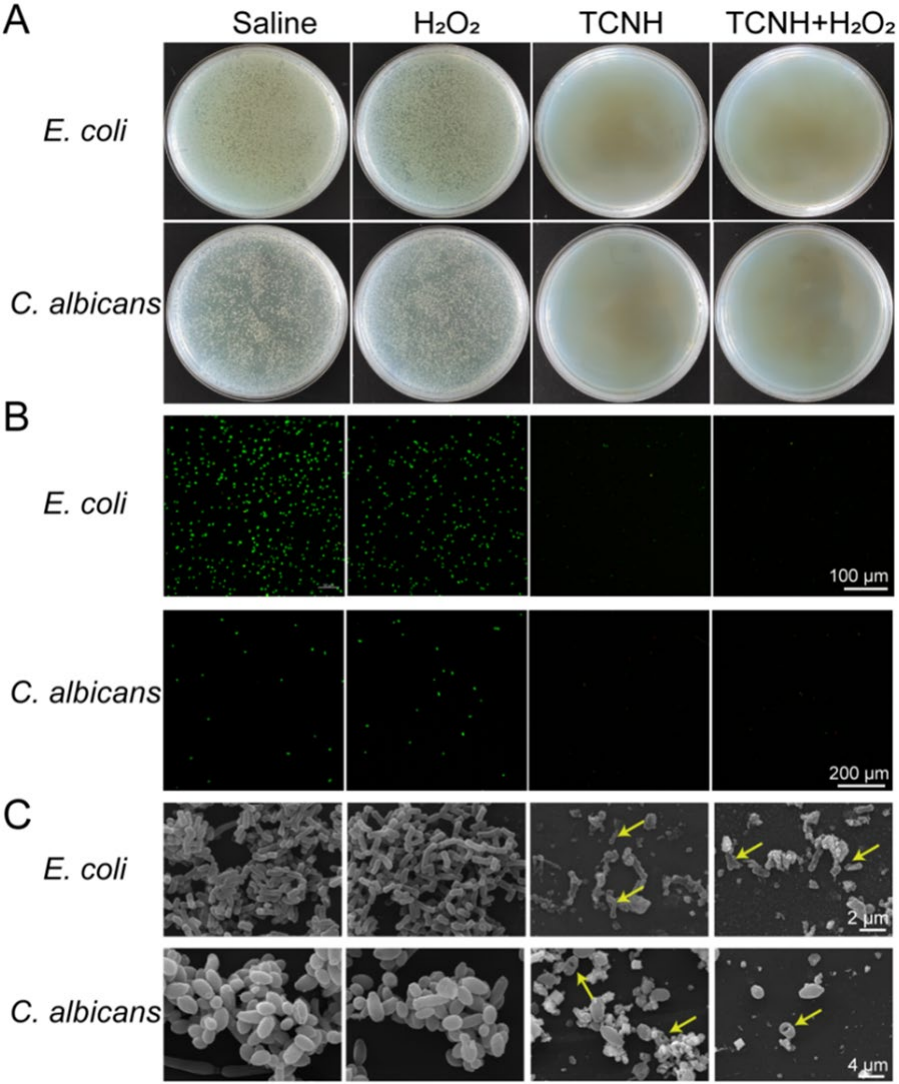
In vitro antibacterial activities of TCNH on *E. coli* and *C. albicans*. Representative photographs of bacterial colonies **A**, CLSM images of live/dead bacteria **B** and SEM images **C** of *E. coli* and *C. albicans* treated with saline, H_2_O_2_, TCNH, or TCNH + H_2_O_2_

In addition, plate cultivation assays (Fig. 6A), dead/live (SYTO 9/PI) double staining experiments (Fig. 6B) and SEM (Fig. 6C) were also adopted to assess the efficacy of TCNH in inhibiting *E. coli* (25922) and *C. albicans* (98001). The absence of bacterial colonies, the decreased numbers of live bacteria and the deformed bacterial cells clarified the broad-spectrum antibacterial activity of TCNH.

### In vivo prophylactic treatment of *P. aeruginosa* keratitis

**Fig. 7:**
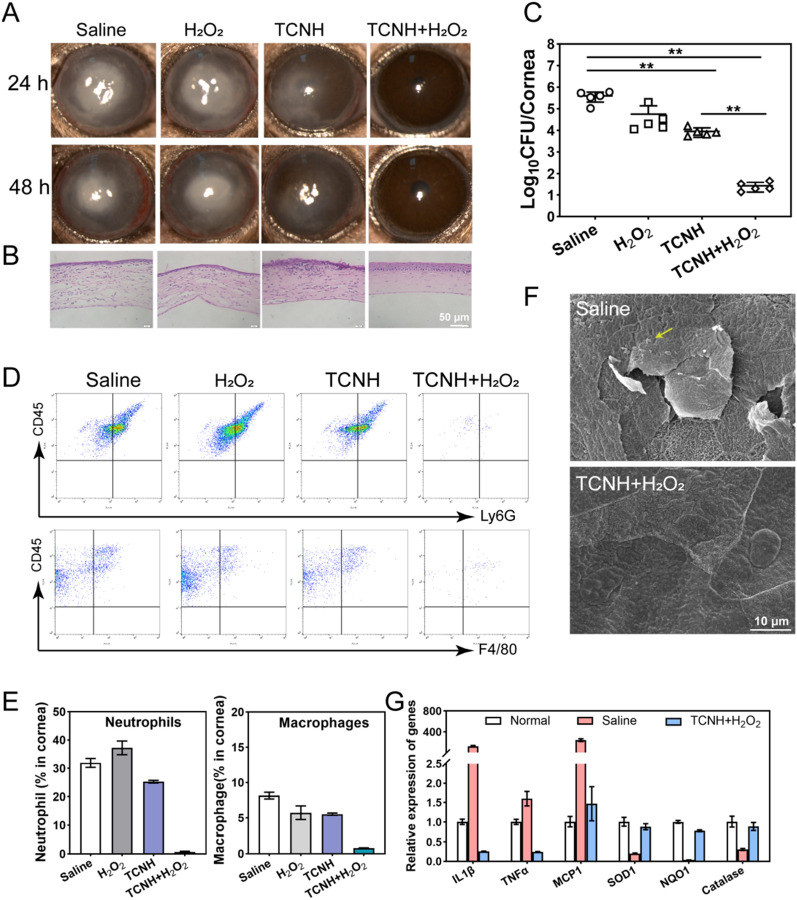
In vivo antibacterial activity of TCNH on *P. aeruginosa* (19660) keratitis. Representative photographs of treated eyes **A**, H&E staining images of mouse corneas **B**, bacterial loads of treated eyes **C**, and the proportions of neutrophils and macrophages in mouse corneas **D**, **E** after saline, H_2_O_2_, TCNH, and TCNH + H_2_O_2_ interventions. Representative SEM images of mouse corneas in the saline and TCNH + H_2_O_2_ groups **F**

The effect of TCNH on prophylactic treatment of *P. aeruginosa* infection was surveyed in vivo using mouse keratitis as the animal model. The corneas with three 1-mm incisions were inoculated with *P. aeruginosa* (19660)*.* Saline, H_2_O_2_, TCNH and TCNH + H_2_O_2_ were dripped on the mouse corneas. At 24 and 48 h post-operation, the mouse corneas were observed using a slit lamp. As shown in Fig. 7A, after intervention with TCNH + H_2_O_2_, the mouse corneas were clear, and no obvious infection was found, which was different from the severe corneal infections in the saline and H_2_O_2_ groups as well as the mild corneal infection in the TCNH group. The corneal samples in the four groups were further subjected to H&E staining. As presented in Fig. 7B, inflammatory cell aggregation was observed in the saline, H_2_O_2_, and TCNH groups. However, no obvious inflammatory cell aggregation was observed in the TCNH + H_2_O_2_ group. The corresponding corneal bacterial loads of *P. aeruginosa* in the four groups were counted. The bacterial number in the TCNH + H_2_O_2_ group was significantly lower than those in the saline, H_2_O_2_, TCNH groups (Fig. 7C). Neutrophils and macrophages of corneas in the saline, H_2_O_2_, TCNH, and TCNH + H_2_O_2_ groups were further sorted by a flow sorter (Fig. 7D, E). The TCNH + H_2_O_2_ group possessed significantly lower proportions of neutrophils (0.63% ± 0.32%) and macrophages (0.80% ± 0.04%) than the other groups. Similar phenomenon can also be observed by immunostaining analysis of macrophages and neutrophils in the four groups (Additional file 1: Figure S5). Furthermore, the corneas in the saline and TCNH + H_2_O_2_ groups were examined by SEM (Fig. 7F). The images showed that the complete and regular morphologies of corneal epithelial cells in the TCNH + H_2_O_2_ group, which was similar with those of normal corneal epithelial cells (Fig. 4E). In contrast, corneal epithelial cells in the saline group tended to slough off the cornea, and the shed cells were always accompanied by *P. aeruginosa* (Fig. 7F, yellow arrow).qRT–PCR was adopted to detect the expression levels of inflammatory factors (IL-1β, TNFα and MCP1) and antioxidant related enzymes (SOD1, NQO1 and catalase) in corneal epithelial cells among the normal, saline, and TCNH groups (Fig. 7G). The results showed that inflammatory factors and antioxidant related enzymes in the saline group were significantly upregulated and downregulated, respectively, compared with those in the normal group. After TCNH + H_2_O_2_ intervention, there was dramatic decrease in inflammatory factor expression and an increase in antioxidant–related enzymes, suggesting that TCNH + H_2_O_2_ may alleviate inflammation and related oxidative stress injury. These results demonstrated that TCNH + H_2_O_2_ possessed positive activity in prophylactic treatment of *P. aeruginosa* keratitis in mice.

### Antibacterial performance of TCNH against MDR *P. aeruginosa*

**Fig. 8:**
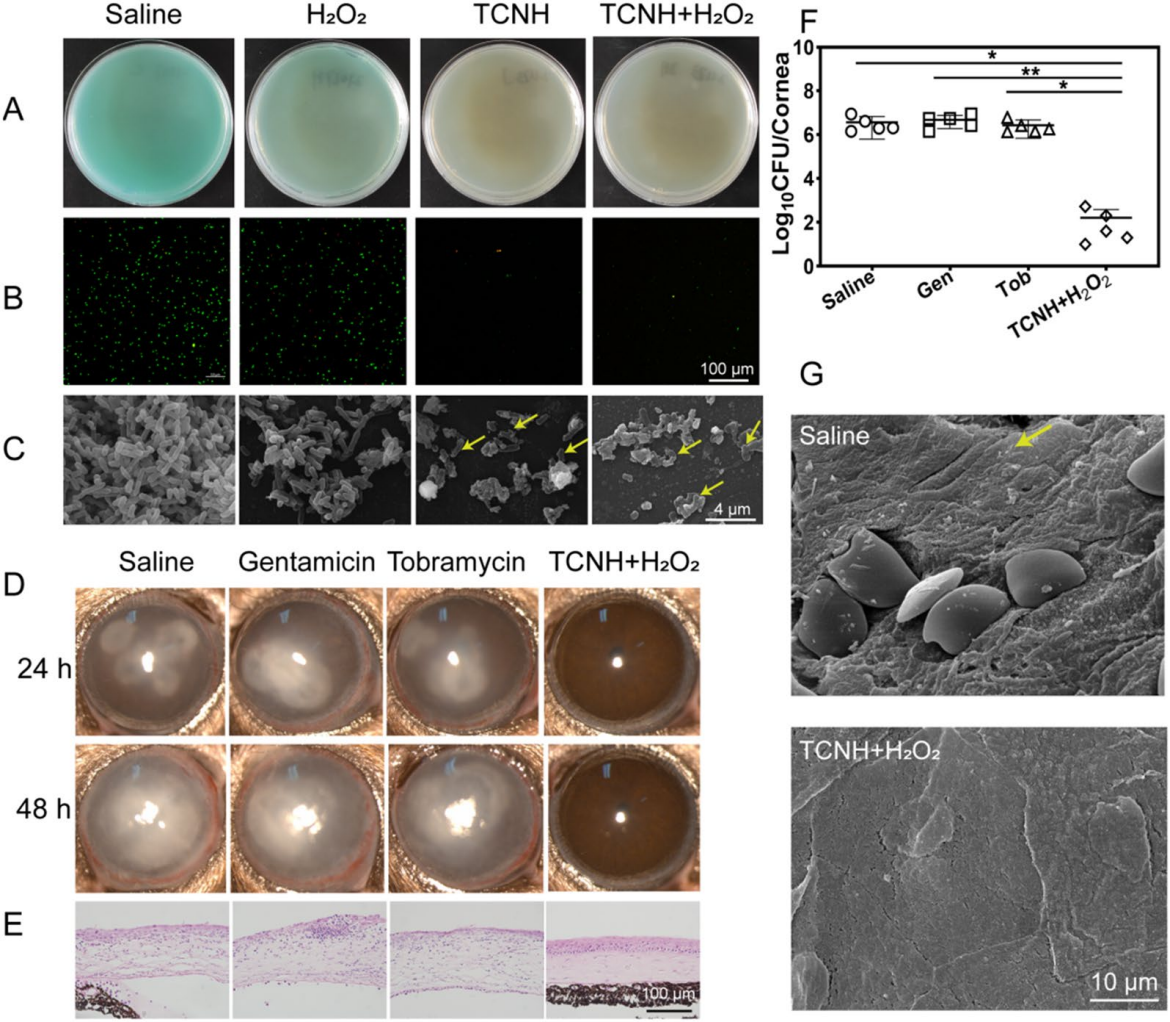
In vitro and in vivo antibacterial activities of TCNH against MDR *P. aeruginosa*. Representative photographs of bacterial colonies **A**, CLSM images of live/dead bacteria **B**, and SEM images of MDR *P. aeruginosa* **C** in the saline, H_2_O_2_, TCNH, and TCNH + H_2_O_2_ groups. Representative photographs **D** and H&E staining images **E**, bacterial loads **F** of treated eyes after saline, H_2_O_2_, TCNH, and TCNH + H_2_O_2_ interventions. Representative SEM images of mouse corneas in the saline and TCNH + H_2_O_2_ groups **G**

Similar with the results of in vitro antibacterial activity against *P. aeruginosa* (19660), there were nearly no MDR bacterial colonies (21027, 20059, 21173, 21715) in the TCNH and TCNH + H_2_O_2_ groups according to the plate cultivation results (Fig. 8A; Additional file 1: Figure S4). Compared with the saline and H_2_O_2_ groups, the numbers of live bacteria (green) in the TCNH + H_2_O_2_ and TCN groups significantly decreased (Fig. 8B), which was also consistent with the plate cultivation results. The bacterial morphology in the four groups was investigated by SEM (Fig. 8C). Deformed *P. aeruginosa* was present in the TCNH + H_2_O_2_ and TCNH groups, while nearly intact *P. aeruginosa* was present in the saline and H_2_O_2_ treated groups, manifesting the in vitro antibacterial efficacies of TCNH and TCNH + H_2_O_2_ against MDR *P. aeruginosa*. Based on the above results, we further inspected the effect of TCNH on prophylactic treatment of MDR *P. aeruginosa* (21027) keratitis with gentamicin and tobramycin as the control groups. After administration of gentamicin and tobramycin, clear corneas and no obvious inflammation were observed in the *P. aeruginosa* (19660) treated group, while similar inflammatory cell infiltration and bacterial loads were observed in the saline (21027) and MDR *P. aeruginosa* (21027) treated groups (Fig. 8D; Additional file 1: Figure S6), which suggested that gentamicin and tobramycin were effective in controlling common strains such as *P. aeruginosa* 19660 other than MDR *P. aeruginosa* strains. Then MDR *P. aeruginosa* infected corneas were cured with TCNH + H_2_O_2_. As a result, there was no inflammatory cell infiltration and an obviously low bacterial load in the corneas (Fig. 8E, F). Furthermore, corneal morphology in the saline and TCNH + H_2_O_2_ groups was also investigated by SEM. The images showed that regular corneal epithelial cells were observed in the TCNH + H_2_O_2_ group, while the detached epithelial cells and MDR *P. aeruginosa* coexisted in the saline group (Fig. 8G, yellow arrow). These results demonstrated the satisfactory prophylactic therapeutic efficacy of the nanozyme composite hybrid hydrogel against MDR *P. aeruginosa* keratitis in mice.

## Conclusions

In this study, tannin-coordinated nanozyme-based hybrid hydrogel eye drops were constructed by photoinitiated free radical polymerization of acetylated gelatin for incorporation of a self-prepared tannin-coordinated nanozyme into a gelatin hydrogel. The hybrid hydrogel exhibited good dispersibility, peroxidase-like activity, biocompatibility, and broad-spectrum antibacterial activity. Due to the intrinsic and nanozymic activities, the composite was successfully used to control *P. aeruginosa* and MDR *P. aeruginosa* keratitis. This was the first time for prophylactic treatment of *P. aeruginosa* infection in vivo and ophthalmic infection using nanozyme. Moreover, many efforts should be made to conduct the clinical prophylaxis of *P. aeruginosa* keratitis using nanozyme hydrogel eye drops. The developed nanozyme hydrogel eye drops provide a promising strategy for the treatment of bacterial and fungal infectious diseases.

## Supplementary Information

**Additional file 1: Figure S1. **SEM of TCNH. **Figure S2.** EDS of TCN. **Figure S3. **Optimization of the H_2_O_2_ and TCNH concentrations. **Figure S4. **In vitro antibacterial activity of TCNH against MDR *P. aeruginosa* (20059, 21173, 21715). Representative photographs of bacterial colonies after intervened by saline, H_2_O_2_, TCNH, and TCNH+H_2_O_2_. **Figure** **S5.** Immunostaining analysis of macrophages and neutrophils in mouse corneas after saline, H_2_O_2_, TCNH, and TCNH+H_2_O_2_ interventions. **Figure S6. **In vivo antibacterial activities of gentamicin and tobramycin on *P. aeruginosa* (19660) keratitis. Representative photographs (A) and hematoxylin–eosin staining images (B) of treated eyes and bacterial loads of treated mouse eyes (C) after gentamicin and tobramycin interventions.

## Data Availability

All data generated or analyzed during this study are included in this published article.
