# Supplementary material for: Tannin coordinated nanozyme composite-based hybrid hydrogel eye drops for prophylactic treatment of multidrug-resistant Pseudomonas aeruginosa keratitis
Source: J Nanobiotechnology. 2022 Oct 14;20:445. doi: 10.1186/s12951-022-01653-w (PMC9563483; doi:10.1186/s12951-022-01653-w)

Supporting Information

Tannin-coordinated nanozyme composite-based hybrid hydrogel eye drops for prophylactic treatment of multidrug-resistant *Pseudomonas aeruginosa* eratitis

Hongwei Wang^1†^, Fangying Song^1†^, Jing Feng^1^, Xia Qi^1^, Li Ma^1^, Lixin Xie^1^, Weiyun Shi^1*^, and Qingjun Zhou^1*^

This supporting information file includes additional results and information as described in the text of the main article. Including:

**Figure S1.** SEM of TCNH.


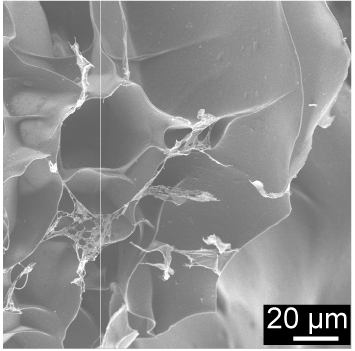


**Figure S2.** EDS of TCN.


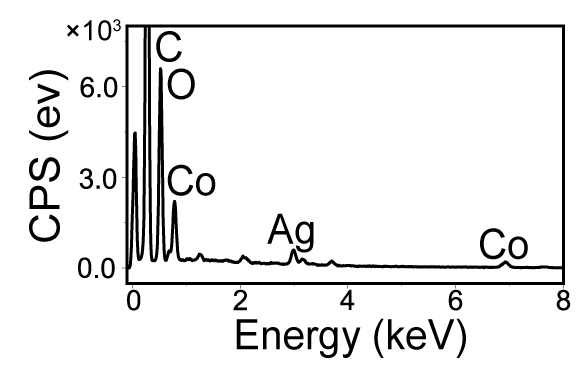


**Figure S3.** Optimization of the H_2_O_2_ and TCNH concentrations


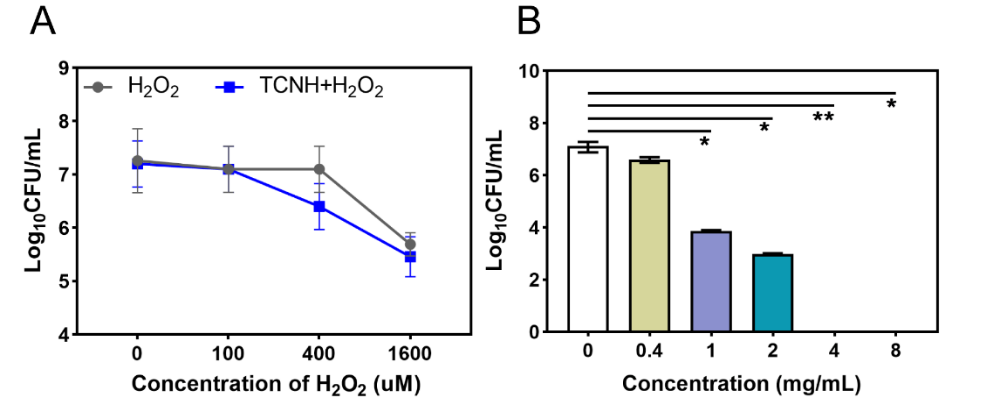


**Figure S4.** In vitro antibacterial activity of TCNH against MDR *P. aeruginosa* (20059, 21173, 21715). Representative photographs of bacterial colonies after intervened by saline, H_2_O_2_, TCNH, and TCNH+H_2_O_2_.


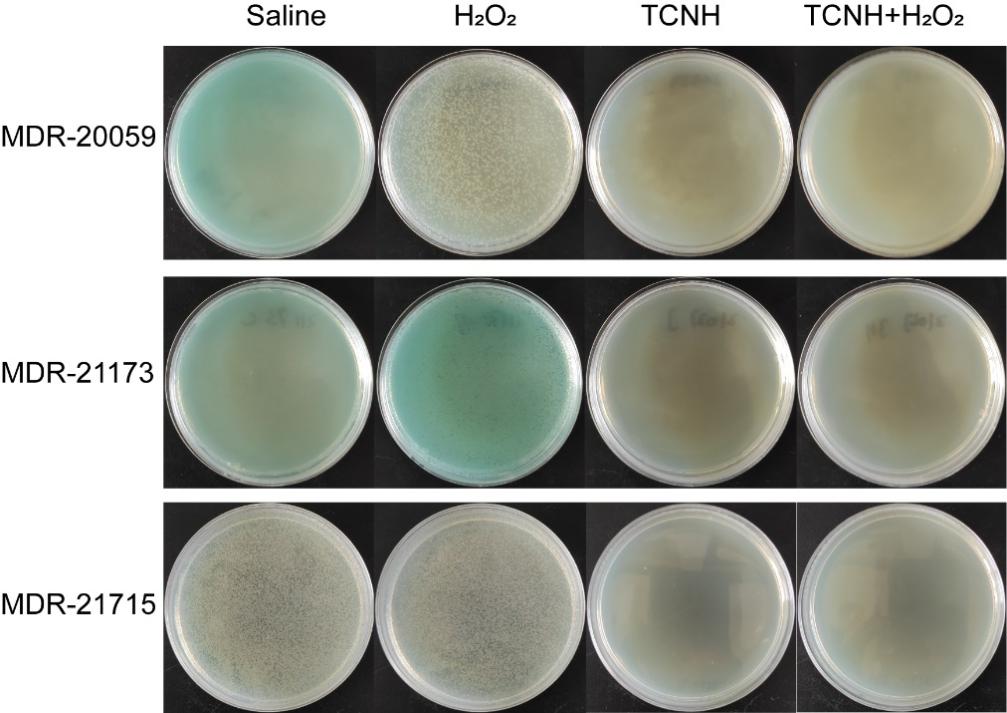


**Figure** **S5.** Immunostaining analysis of macrophages and neutrophils in mouse corneas after saline, H_2_O_2_, TCNH, and TCNH+H_2_O_2_ interventions.


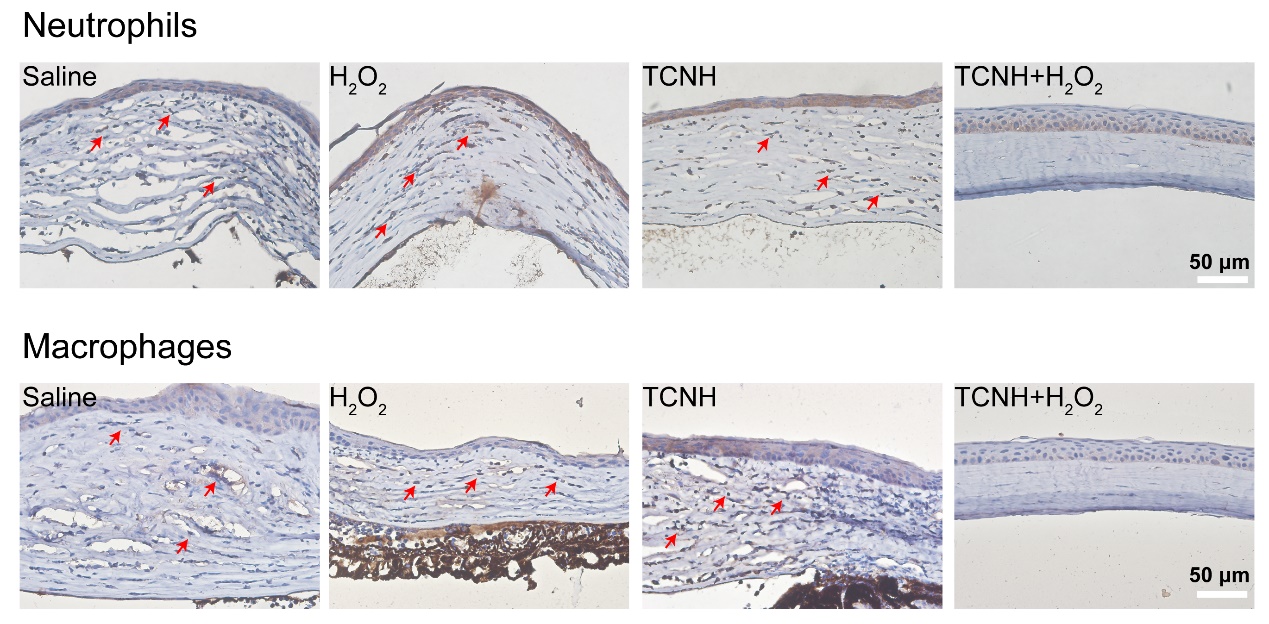


**Figure S6.** In vivo antibacterial activities of gentamicin and tobramycin on *P. aeruginosa* (19660) keratitis. Representative photographs (A) and hematoxylin–eosin staining images (B) of treated eyes and bacterial loads of treated mouse eyes (C) after gentamicin and tobramycin interventions.


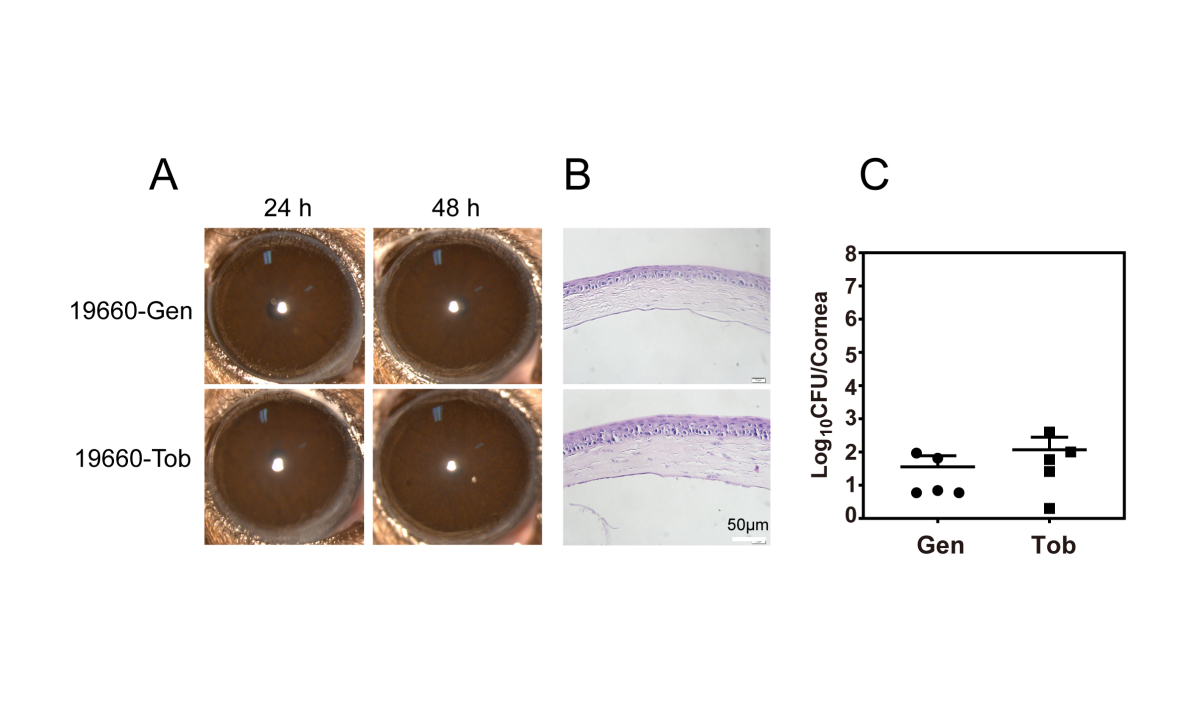

Supplement: Supplementary file 1 — Additional file 1: Figure S1. SEM of TCNH. Figure S2. EDS of TCN. Figure S3. Optimization of the H2O2 and TCNH concentrations. Figure S4. In vitro antibacterial activity of TCNH against MDR P. aeruginosa (20059, 21173, 21715). Representative photographs of bacterial colonies after intervened by saline, H2O2, TCNH, and TCNH+H2O2. Figure S5. Immunostaining analysis of macrophages and neutrophils in mouse corneas after saline, H2O2, TCNH, and TCNH+H2O2 interventions. Figure S6. In vivo antibacterial activities of gentamicin and tobramycin on P. aeruginosa (19660) keratitis. Representative photographs (A) and hematoxylin–eosin staining images (B) of treated eyes and bacterial loads of treated mouse eyes (C) after gentamicin and tobramycin interventions. [file 12951_2022_1653_MOESM1_ESM.docx]
